# Humanization of Yeasts for Glycan-Type End-Products

**DOI:** 10.3389/fmicb.2022.930658

**Published:** 2022-07-07

**Authors:** Xingjuan Li, Jianlie Shen, Xingqiang Chen, Lei Chen, Shulin Wan, Xingtao Qiu, Ke Chen, Chunmiao Chen, Haidong Tan

**Affiliations:** Guangzhou Shenjingya Agricultural Technology Co., Ltd., Guangzhou, China

**Keywords:** humanized glycosylation, yeasts, genetically engineering, end-products, galactosylated complex-type glycans

## Abstract

Yeasts are often considered microorganisms for producing human therapeutic glycosylated end-products at an industrial scale. However, the products with non-humanized glycans limited their usage. Therefore, various methods to develop humanized glycosylated end-products have been widely reported in yeasts. To make full use of these methods, it is necessary to summarize the present research to find effective approaches to producing humanized products. The present research focuses on yeast species selection, glycosyltransferase deletion, expression of endoglycosidase, and expression of proteins with galactosylated and or sialylated glycans. Nevertheless, the yeasts will have growth defects with low bioactivity when the key enzymes are deleted. It is necessary to express the corresponding repairing protein. Compared with N-glycosylation, the function of yeast protein O-glycosylation is not well-understood. Yeast proteins have a wide variety of O-glycans in different species, and it is difficult to predict glycosylation sites, which limits the humanization of O-glycosylated yeast proteins. The future challenges include the following points: there are still many important potential yeasts that have never been tried to produce glycosylated therapeutic products. Their glycosylation pathway and related mechanisms for producing humanized glycosylated proteins have rarely been reported. On the other hand, the amounts of key enzymes on glycan pathways in human beings are significantly more than those in yeasts. Therefore, there is still a challenge to produce a large body of humanized therapeutic end-products in suitable yeast species, especially the protein with complex glycans. CRISPR-Cas9 system may provide a potential approach to address the important issue.

## Introduction

Engineering Escherichia coli has been widely used for the production of human advanced biopharmaceutical products (Arico et al., 2013). However, the recombinant proteins in non-glycosylated forms often suffer from short-term half-life, low activity, rapid clearance, and side effects. Pharmacokinetic studies in Wistar rats revealed 1.3-fold increase in plasma half-life for glycosylated IFN α2b compared to standard IFN α2b produced by *E. coli* (Baghban et al., 2018). Therefore, it is necessary to develop humanized glycosylated therapeutic products (Cheon et al., 2012). Yeasts are often firstly considered microorganisms for producing human therapeutic glycosylated end-products at an industrial scale. Long historical usage, vast data, and rich experiences have paved the way for *Saccharomyces cerevisiae* as an important expression platform and a microbial cell factory for producing human therapeutic products (Corbacho et al., 2005). In *S. cerevisiae*, the recombinant proteins are often typically hypermannosylated (Man_>50_GlcNAc_2_), which also affects the half-life, tissue distribution, and immunogenicity of the end-products. It is critical to developing the engineering of *S. cerevisiae* for producing the proteins with humanized glycosylation patterns by modulating mannosyltransferase and or other enzymes responsible for the chain biosynthesis of N-glycans (Crauwels et al., 2015).

*Pichia pastoris* expression system is one of the most popular tools for producing a recombinant protein with many advantages, including the low incidence of hyperglycosylated, appropriate folding in the endoplasmic reticulum (ER), and secretion expression with high purity (De Pourcq et al., 2012). Much effort has been paid to improving the N-glycosylation pathway of *P. pastoris* to mimic the human N-glycosylation pathway. Successful utilization of the CRISPR-Cas9 system has been used for the humanization of the glycosylation pathways in *P. pastoris* by disrupting och1 and alg3 (mannosyltransferase) genes (Dolashka-Angelova et al., 2010). *Kluyveromyces lactis* is a food-grade yeast commonly used for industrial applications. Gene modification has been applied to construct a humanized N-linked glycosylation pathway in *K. lactis*. Besides the above conventional yeast species, other established yeast expression systems include *Hansenula polymorpha* (Gündüz Ergün et al., 2019; Fukunaga et al., 2021a,b; Giorgetti et al., 2021; Gallo et al., 2022), *Yarrowia lipolytica* (Hamilton et al., 2013; Karbalaei et al., 2020; Jin et al., 2021), *Schizosaccharomyces pombe* (Kim et al., 2006; Katla et al., 2019; Lawton et al., 2021), *Kluyveromyces marxianus* (Lee et al., 2020), *Candida albicans* (Li et al., 2019), and *Zygosaccharomyces cidri* (Liu et al., 2009). The strategies to produce humanized glycosylated end-products were explored by using these yeast species.

## N-Glycosylated Forms of Proteins in Yeasts and Mammalian Cells

**Figure 1:**
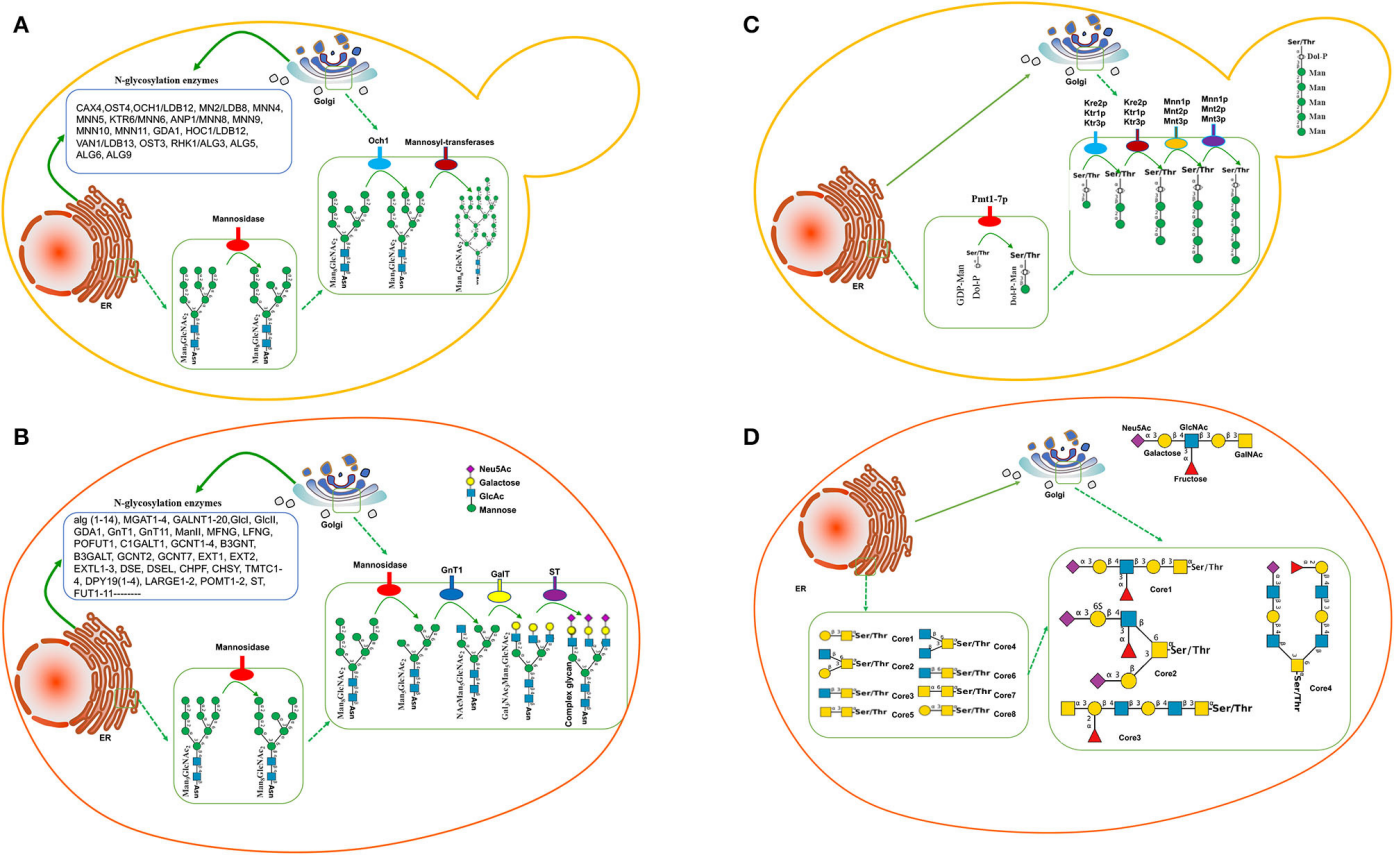
Glycosylation pathway between yeasts and mammalian cells. **(A)** N-glycosylation pathway in yeasts. **(B)** N-glycosylation pathway in mammalian cells. **(C)** O-glycosylation pathway in *Saccharomyces cerevisiae*. In the ER, the first mannose residue is added *via* protein-O-mannosyltransferases (Pmt1-7p). O-glycosylation is further elongated in the Golgi *via* several mannosyltransferases. **(D)** Mucin-type O-glycan synthesis in human cells.

The N-glycosylation has been widely studied in yeast species, indicating that the glycosylated signaling pathway is similar to that in mammalian cells. Man_3_GlcNAc_2_ is the common core of eukaryotic N-glycans, which further yields the Man_8_GlcNAc_2_ core *via* oligosaccharyltransferase. Man_8_GlcNAc_2_-Asn-protein is produced in the endoplasmic reticulum (ER) and conveyed to Golgi (Figure 1A). In Golgi, there is a significant difference in the glycosylation formation of proteins, Man_8_GlcNAc_2_ glycan is truncated into Man_5_GlcNAc_2_ *via* mannosidase in mammalian cells (Figure 1B) while Man_8_GlcNAc_2_ in yeasts is extended to form Man_9_GlcNAc_2_ and hypermannosylation *via* mannosyl transferase (Figure 1A), which will be cleared in human blood because of its non-human glycan (Ma et al., 2019). Genome-wide analysis for *S. cerevisiae* shows 19 different genes associated with mannoprotein-linked oligosaccharide generation, including CAX4 (dolichyl pyrophosphate (Dol-PP) phosphatase), OST4 (oligosaccharyltransferase), OCH1/LDB12 (Mannosyltransferase), MNN(Mannosyltransferase)2/LDB8, MNN4, MNN5, KTR6/MNN6, ANP1/MNN8, MNN9, MNN10, MNN11, GDA1 (Golgi GDPase), HOC1/LDB12 (glycosyltransferase), VAN1/LDB13 (glycosylation of secreted invertase), OST3, RHK1/ALG(mannosyltransferase)3, ALG5, ALG6, and ALG9, which mainly existed in nucleic ER and Golgi ER (Figure 1A; Narimatsu et al., 2019). Most yeast species produce the glycosylated protein with mannose units with long chains.

Comparatively, the transferases in mammalian cells are significantly more than those in *S. cerevisiae*. More than 170 human glycosyltransferases have been found in the glycosylation pathway (Figure 1B; Oh et al., 2008). Most mammals produce the glycosylated protein with the mannose, galactose, and sialic acid N-glycolylneuraminic acid (Neu5Gc) units in the short-chain (Figure 1B). Therefore, the glycosylated proteins in yeast species are significantly longer than those in mammalian cells while the ingredients in mammalian cells are more complex than those in yeast species.

## O-Glycosylated Forms of Proteins in Yeasts and Mammalian Cells

Compared with N-glycosylation, the function of yeast protein O-glycosylation is poorly understood. Yeast proteins have a wide variety of O-glycans in different species, and it is difficult to predict glycosylation sites, which limits the humanization of O-glycosylated yeast proteins. The first mannose linked to yeast protein is provided by polyterpenoid phosphate mannose (Dol-P-Man). Dol-P-Man is synthesized from guanosine diphosphate-mannose (GDP-Man) and polyterpenoid phosphate mannose (Dol-P) under the catalysis of Dol-P-Man synthase, and then, enters the endoplasmic reticulum to initiate protein O-glycosylation (Figure 1C). In contrast, mucin-type O-glycan synthesis in human cells starts with an N-acetylgalactosamine (GalNAc) residue attached with specific Ser/Thr residues with eight-type cores. The initiating residues are further elongated with a variety of monosaccharides. In the Golgi apparatus, the elongation of oligosaccharides is catalyzed by GalNAc transferases using UDP-GalNAc as a donor (Figure 1D). However, manufacturing the O-glycosylation pathway is not as progressive as N-glycosylation. Humanized mucin-type O-glycans had been identified in *S. cerevisiae* to provide a biosynthetic pathway for UDP-GalNAc and UDP-Gal/GalNAc-4-epimerase production (Luu et al., 2019).

## An Exploration of Strategies to Regulate Glycosylation of End-Products in Yeasts

Although yeasts have the potential to produce various types of industrial proteins, their failures to produce humanized glycoproteins limit their usage. Therefore, it is necessary to explore the strategies to regulate glycan forms of end-products in yeasts.

### Deletion of Mannosyltransferase

The glycan chains in yeast species are often longer than those in human beings. To fix the flaw, the key enzyme mannosyltransferase, which existed in N-glycosylation pathways, is often considered to be blocked. Och1(α-1,6-mannosyltransferase) and MNN1 (α-1,3-mannosyltransferase) produce mostly high-mannose glycans of the Man8GlcNAc2 (Figure 1A). Inactivation of the OCH1 gene is often an effective approach to eliminate hypermannosylation in *H. polymorpha* (Fukunaga et al., 2021b; Gallo et al., 2022), *K. lactis* (Park et al., 2011), *P. pastoris* (Ma et al., 2019), *S. cerevisiae* (Piirainen et al., 2022), and *Y. lipolytica* (Karbalaei et al., 2020), which results in the expressed proteins with short-chain glycan from Man_3_GlcNAc_2_ to Man_14_GlcNAc_2_ (Table 1). Especially in *H. polymorpha*, the deletion of OCH1 and alg3, and/or alg3 and alg11 can produce the protein with Man_3_GlcNAc_2_ (Gündüz Ergün et al., 2019; Fukunaga et al., 2021b). The expression of alg6 in *S. pombe* also produces the short-chain glycoprotein (Hamilton et al., 2013).

**Table 1:** Humanized glycoprotein expression in genetically modifying yeasts or naturally yeasts.

**Yeast species**	**Δ Glycosyltransferases (overexpression of enzyme)**	**End-products and manosylated types**			**References**
		**Expressed protein**	**Wild species**	**Mutant species**	
*Hansenula polymorpha*	OCH1, alg3(GnT1)	α-1,2 mannosidase		GlcNAc_1_Man_5_GlcNAc_2_ GlcNAc_1_Man_3_GlcNAc_2_	Fukunaga et al., 2021a
	OCH1, alg3	α-1,2 mannosidase		Man_3_GlcNAc_2_	Fukunaga et al., 2021b
	OCH1, OCR1	α-1,2 mannosidase	Man_8_GlcNAc_2_	Man_5_GlcNAc_2_	Gallo et al., 2022
	alg3, alg11(GnTI, GnTII, GalTI)			Gal_2_GlcNAc_2_Man_3_GlcNAc_2_	Giorgetti et al., 2021
	alg3, alg11	Glucose oxidase		Man_3_GlcNAc_2_	Gündüz Ergün et al., 2019
*Kluyveromyces cicerisporus*		Exo-inulinase		Man_3−9_GlcNAc_2_	Vervecken et al., 2004
*Kluyveromyces lactis*	OCH1, MNN1	HAS, GM–CSF	Man_>30_GlcNAc_2_	Man_9−11_GlcNAc_2_, Man_13−14_GlcNAc_2_	Wang et al., 2013
	KlOCH1	HAS, GAA		Shorter β1,6-glucan and more branches	Park et al., 2011
	KlPMR1	HAS	HGS	NHGS	Wang et al., 2020
*Kluyveromyces marxianus*		Cu/Zn SOD	Man_5_GlcNAc_2_		Lee et al., 2020
*Pichia pastoris*	OCH1 (MS/AT/GT)		Up to Man_40_GlcNAc_2_	Man_5_GlcNAc_2_ GlcNAcMan_5_GlcNAc_2_	Ma et al., 2019
	(Endo-T)	IgG1-Fc	Up to Man_40_GlcNAc_2_	*N*-GlcNAc	Prill et al., 2005
	Glycoengineered strain	IFN α2b		SuperMan5	Baghban et al., 2018
	PMT1, PMT5	TG		Man1_2_GlcNAc_2_~ Man16GlcNAc_2_	Watanasrisin et al., 2016
	alg3, alg11(GTFU)	CWP and SP		Man_3_GlcNAc_2_ GNG	Selas Castiñeiras et al., 2018
*Saccharomyces cerevisiae*	(AST)			SOG	Song et al., 2010
	(Endos2)	Fc γ receptors		SNR	Ryckaert et al., 2005
	OCH1, MNN1, alg3			Man_5_GlcNAc_2_ Man_8_GlcNAc_2_	Piirainen et al., 2022
	Pmd1 null mutant	P-glycoprotein		No glycosylation	Xu et al., 2017
*Schizosaccharomyces pombe*	OCH1 (ST)			Glycan with sialic acid	Shenoy et al., 2021
			3 Glc and 9 Man	5 Man	Katla et al., 2019
	Gmn2			DPGNO	Kim et al., 2006
	Gmh1p, Gmh2p Gmh3p, Gmh6p			Downregulated galactosylation	Lawton et al., 2021
*Yarrowia lipolytica*	(alg6)	α-1,2-mannosidase		Man_3_GlcNAc_2_	Hamilton et al., 2013
	MPO1	CWPs			Jin et al., 2021
	OCH1	Lipase	Man_12_GlcNAc_2_	Man_8_GlcNAc_2_	Karbalaei et al., 2020

*alg, asparagine-linked glycosylation protein; Complex glycan, the glycan with galactose and sialic acid; AST, β-1,2-N-acetylglucosaminyltransferase 1; CWPs, crude cell wall mannoproteins; Cu/Zn SOD, Cu/Zn-superoxide dismutase; CWP and SP, cell wall proteins and secreted proteins; DPGNO, defects in protein glycosylation of N-linked oligosaccharides; EndoS2, An IgG-specific endoglycosidase; Endo-T, ENGase isoform which possesses powerful hydrolytic activities toward high-mannose type N-glycans; FTVI, α-1, 3-fucosyltransferase VI; GAA, glucoamylase; Gal10, UDP-glucose 4-epimerase; GalTI, β-1,4-galactosyltransferase I; GM–CSF, granulocyte–macrophage colony-stimulating factor fusion protein; Gmh1p, Gmh2p, Gmh3p, and Gmh6p, α1,2-galactosyltransferases and α1,3-galactosyltransferases; GNG, galactosylated N-glycans complex; GnTI, β-1,2-N-acetylglucosaminyltransferase I; GnTII, β-1,2-N-acetylglucosaminyltransferase II; GTFU, a human galactosyltransferase fused protein with a UDP-glucose 4-epimerase domain from Schizosaccharomyces pombe; HAS, human serum albumin; HGS, hyperglycosylated protein; KlOCH1, β1,6- mannosyltransferase; KlPMR1, a Ca2+-ATPase localized in the Golgi apparatus involved in the glycosylation and cell wall morphogenesis; MNT, a-1,2-Mannosyltransferase; MNN1, α1,3-mannosyltransferase; MPO1, MNN4 homolog; MS/AT/GT, α-1,2-mannosidase, N-acetylglucosaminyltransferase I and β-1,4-galactosyltransferase; OCH1, α1,6-mannosyltransferase; NHGS, non-hyperglycosylated protein; PMD1, leptomycin efflux transporter Pmd1; PMT1 and PMT5, O-mannosyltransferase; P-glycoprotein, the transporters determine the uptake and efflux of drugs; SOG, sialic acid-O-linked glycan; ST, sialyltransferase; SNR, a single N-acetylglucosamine residue; TG, therapeutic glycoproteins*.

### Overexpression of Repairing Proteins

Protein glycosylation is often associated with cell wall integrity and its disruption may affect yeast bioactivity. The yeasts will have growth defects and low bioactivity when the key enzymes are deleted. Inactivation of the OCH1 gene will damage *S. cerevisiae* viability. The overexpression of glycosylphosphatidylinositol (GPI)-anchored protein can restore the bioactivity (Table 1; Prabhakar et al., 2019). Therefore, overexpression of repairing proteins may be an effective approach to improve the yields of humanized products.

### Overexpression of Endoglycosidase

The overexpression of endoglycosidase α-1,2-mannosidase can prevent the glycan chain from further extending in *P. pastoris* (Ma et al., 2019). ENGase isoform possesses great hydrolytic activities toward high-mannose N-glycans and short-chain glycan was produced after the ENGase was expressed in *P. pastoris* (Prill et al., 2005). EndoS2, an IgG-specific endoglycosidase, also has a similar function to ENGase and was expressed in *S. cerevisiae* to produce a single N-acetylglucosamine residue (Ryckaert et al., 2005).

### Production of Galactosylated Protein

In mammalian cells, GlcNAc sugars are attached to the paucimannose core with 2-4 GlcNac sugars, which can be further prolonged by adding galactose *via* GalTI (β-1,4-galactosyltransferase I). GalTI was expressed in *H. polymorpha* and increased the formation of galactosylated complex-type glycans (Giorgetti et al., 2021). A human galactosyltransferase fused protein with a UDP-glucose 4-epimerase domain from *S. pombe* was expressed in *P. pastoris* and also produced galactosylated proteins (Selas Castiñeiras et al., 2018).

### Production of Sialylated Glycoproteins

The lack of sialylation on glycoprotein will reduce its efficacy as a therapeutic agent because of the rapid clearance of the protein from the human bloodstream. Human glycans are often capped terminally with sialic acid *via* sialyltransferase (ST, localized in Golgi, Figure 1B). A more effective biocatalyst system for producing sialylated glycoproteins was established in *S. cerevisiae via* the deletion of OCH1 and expression of ST more than one decade ago (Shenoy et al., 2021). Sialylated O-linked glycans were produced in *P. pastoris via* the expression of β-1,2-N-acetylglucosaminyltransferase 1 (AST; Song et al., 2010).

### Selection of Yeast Species

Among all the selected yeast species, *H. polymorpha, K. marxianus, S. pombe*, and *Y. lipolytic* tend to produce short-chain-glycan proteins than other yeast species (Table 1). Therefore, these yeast species may be more suitable to produce humanized end-products.

## Discussion

Over the past decades, engineering modification of N-glycosylation pathways in yeast species has developed significantly. Most yeast strains are suitable to produce the end-products with hybrid N-glycosylation, galactosylation, and sialyation complex glycans at high uniformity (Table 1). With the development of the yeast expression system, it is expected that yeast strains with the ability to generate fully humanized proteins or end-products will be attainable shortly. Nonetheless, there are still some challenges in exploring the yeast expressing system.

- *Arxula adeninivorans* is an unconventional, non-pathogenic, and haploid yeast and maybe a new expression platform (Song et al., 2007). *Brettanomyces bruxellensis*, a wine yeast, mainly contributes to spontaneous beer fermentations associated with industrial fermentation ecosystems (Tanaka et al., 2021). *Brettanomyces claussenii*, a yeast often found on pineapple or other tropical fruits, expands the potential for the valorization of dairy by-products to functional beverages (Turakainen et al., 1994). *Candida utilis* is a commercial food additive and a potentially beneficial host for producing heterologous protein (Uccelletti et al., 2004). *Hanseniaspora uvarum* (anamorph *Kloeckera apiculata*) is an apiculate yeast species normally found on wine grapes and other fruits and has a strong influence on wine quality. *Zygosaccharomyces bailii* is a yeast species widely present in wine, tea, and vinegar fermentations (Uccelletti et al., 2006). However, these yeasts, and their N-glycosylation pathway and related mechanisms for producing humanized glycosylated proteins have rarely been reported.
- There are more than 20 glycosyltransferases found in the glycosylation pathway in *S. cerevisiae* (Figure 1A; Narimatsu et al., 2019) and 170 human glycosyltransferases found in the glycosylation pathway (Figure 1B; Oh et al., 2008). Therefore, there is still a challenge to produce a large body of humanized therapeutic end-products with complex glycan in suitable yeasts. Making full use of the CRISPR-Cas9 technique may be a potential strategy to address the important issues (Ueda et al., 1993; Dolashka-Angelova et al., 2010).

## Author Contributions

XL, XC, LC, SW, and CC collected and analyzed all data. XQ, XL, XC, JS, KC, and HT contributed to the conception and designing the article and interpreted the relevant literature. XL and HT drafted and revised the article. The manuscript has been read and approved by all authors before submission.

## Funding

The present work was supported by Guangzhou Zengcheng Qiaomengyuan Branch Project (Grant No. 20210300X).

## Conflict of Interest

XL, JS, XC, LC, SW, XQ, KC, CC, and HT were employed by Guangzhou Shenjingya Agricultural Technology Co., Ltd.

## Publisher's Note

All claims expressed in this article are solely those of the authors and do not necessarily represent those of their affiliated organizations, or those of the publisher, the editors and the reviewers. Any product that may be evaluated in this article, or claim that may be made by its manufacturer, is not guaranteed or endorsed by the publisher.
